# Complete mitochondrial genome of the Greenland wolf, *Canis lupus orion*

**DOI:** 10.1080/23802359.2019.1660594

**Published:** 2019-09-02

**Authors:** Hyunjun Cho, Bo-Mi Kim, Won Young Lee, Jae-Sung Rhee

**Affiliations:** a Division of Polar Life Sciences, Korea Polar Research Institute, Incheon, South Korea;; b Unit of Polar Genomics, Korea Polar Research Institute, Incheon, South Korea;; c Department of Marine Science, College of Natural Sciences, Incheon National University, Incheon, South Korea;; d Research Institute of Basic Sciences, Incheon National University, Incheon, South Korea

**Keywords:** Greenland wolf, *Canis lupusorion*, gray wolf, mitogenome

## Abstract

The Greenland wolf, *Canis lupus orion* as s subspecies of the gray wolf, is native to Greenland. Here, we assembled a complete 16,650 bp genome for the *C. l. orion* mitochondrion by employing Illumina HiSeq platform. The complete mitochondrial genome contained 13 protein-coding genes (PCGs), 22 transfer RNA (tRNA) genes, two ribosomal RNA (rRNA) genes, and one control region. Overall DNA sequence of the *C. l. orion* mitochondrion was identical to that of gray wolf *C. l. lupus*, although slight difference was observed in their control regions. The genomic structure of *C. l. orion* mitochondrion was conserved with the gene arrangements of mitogenomes published in Canidae, and phylogenetic analysis confirmed the sister relationship among *Canis* sp. This information will provide essential molecular reference to elucidate biogeography, phylogenetic distance, and evolutionary history in gray wolves.

The gray wolf (*Canis lupus*) occupies a wide range of geographical distribution with ecological flexibility, ranging from the Middle East to the Holarctic regions. Since the species has complex evolutionary history particularly with coyotes and domestic dogs, extensive DNA information of gray wolves has been phylogenetically employed with evidence of environmental and morphological characteristics (Freedman et al. 2014; Fan et al. 2016; Gopalakrishnan et al. 2018; Sinding et al. 2018). Arctic wolf was firstly described as *C. l. arctos* from the samples of Melville Island and Ellesmere Island of the Canadian Arctic Archipelago (Pocock 1935). Subsequently, two subspecies of the Arctic wolf, *C. l. orion* and *C. l. bernardi* were recognized by Nowak (1995) as synonyms of *C. l. arctos* based on their morphometric characteristics and free movement in the northern Greenland range (Dawes et al. 1986). However, research on the phylogenetic relationship and evolutionary history of *C. l. orion* remains to be still explored due to insufficient genomic information and limited geographic samples. Previous analysis using mitochondrial control region suggested that Greenland samples might be an isolated population by possible colonization from the Canadian Arctic Archipelago (Ersmark et al. 2016). Thus, accumulation of information on the complete mitochondrial genome of Greenland wolves will be helpful to understand molecular phylogeny and genetic diversity of gray wolves.

In this study, we sequenced the complete mitogenome of *C. l. orion* (Accession no. MK948871 contents should be updated in the GenBank system). Hair sample was isolated from a single individual of *C. l. orion*, which had visited our field campsite and left hairs on the tent at Sirius Passet (82°47′4.3″N, 42°27′11.6″W; Lee 2018) on 5th July 2017. The voucher specimen was deposited in the Korea Polar Research Institute (KOPRI; Species ID: GW; Specimen ID: 170705_GW01). Genomic DNA was extracted from hair root by using the DNeasy Blood and Tissue kit (Qiagen, Hilden, Germany). The genomic DNA was quantified using a Qubit 4 Fluorometer (Thermo Fisher Scientific, Inc., Waltham, MA, USA). The library construction and sequencing were performed by a commercial company (Macrogen, Seoul, South Korea). Genomic libraries were constructed from total genomic DNA (1 μg) using the TruSeq RNA Sample Preparation Kit according to the manufacturer's instructions (Illumina, San Diego, CA, USA). The generated raw reads were pre‐processed and adapter sequences, low-quality reads (sequences with >50% bases with quality value ≤5), reads with >10% of unknown bases, and ambiguous bases were totally removed to obtain high quality reads for assembly. To generate contigs, *de novo* assembly was performed using high-quality reads by various k-mer using A5-pipeline. Additional PCR procedure was conducted to confirm the DNA sequence of control region. Overall sequences were annotated by using the MITOS web-based software (Bernt et al. 2013) and detailed annotation were conducted with NCBI-BLAST (http://blast.ncbi.nlm.nih.gov).

The complete mitogenome of *C. l. orion* was 16,650 bp in length and contained the typical set of 13 PCGs, 22 tRNAs, two rRNAs, and one control region, as shown in Carnivora mitogenomes. The nucleotide composition of *C. l. orion* mitogenome is heavily biased toward A + T nucleotides, accounting for 32% A, 25% C, 14% G, and 29% T. Overall gene order and content of *C. l. orion* mitogenome were identical to those of genus *Canis*. The 13 PCGs of *C. l. orion* mitogenome showed similarity with *C. l. lupus* (99.8%), *C. l. familiaris* (99.4%), *C. l. laniger* (96.8%), *C. l. chanco* (96.8%), *C. anthus* (96.6%), and *C. latrans* (94.8%). In addition, significant difference was observed in their control regions possibly due to putative substitutions and/or indels of nucleotides. A phylogenetic analysis was constructed using the concatenated set of 13 PCGs of *C. l. orion* mitogenome with including of 15 published mitogenomes from Canidae (Figure 1). We used JModelTest ver. 2.1.10 (Darriba et al. 2012) to select the best substitution model and a substitution model (HKY + G+I) was employed to construct a maximum-likelihood (ML) method in the PhyML 2.4.5 (Guindon and Gascuel 2003) with 1000 bootstrap replicates. The *C. l. orion* mitogenome was clustered into a clade within *C. lupus* clade and is supported as sister taxa to *C. l. lupus*. In conclusion, the complete *C. l. orion* mitogenome will provide useful information to elucidate phylogenetic relationship, geographical distribution, and evolution of the genus *Canis* and related subspecies.

**Figure 1.**
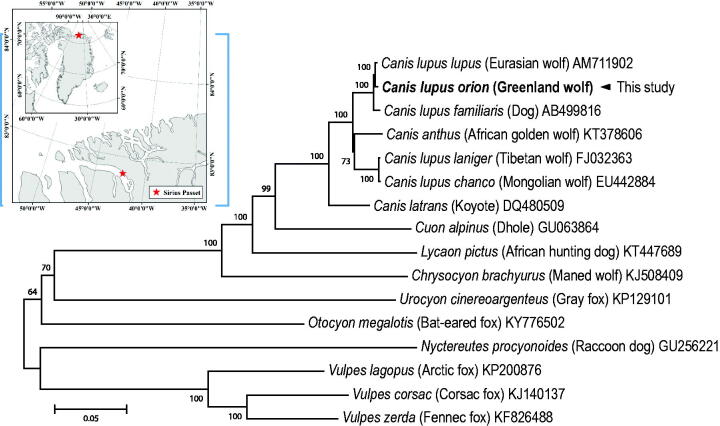
Maximum-likelihood (ML) phylogeny of 16 Canidae species based on the concatenated nucleotide sequences of protein-coding genes (PCGs). Numbers on the branches indicate ML bootstrap percentages (1000 replicates). DDBJ/EMBL/Genbank accession numbers for published sequences are incorporated. Small box represents the sampling site in Greenland.
